# Current practices in library/informatics instruction in academic libraries serving medical schools in the western United States: a three-phase action research study

**DOI:** 10.1186/1472-6920-13-119

**Published:** 2013-09-04

**Authors:** Jonathan D Eldredge, Karen M Heskett, Terry Henner, Josephine P Tan

**Affiliations:** 1 Health Sciences Library & Informatics Center and Department of Family & Community Medicine, University of New Mexico, MSC09 5100, Albuquerque, NM 87131-0001, USA; 2 UC San Diego Biomedical Library, UC San Diego, 9500 Gilman Dr. 0699, La Jolla, CA 92093, USA; 3 Savitt Medical Library, University of Nevada School of Medicine, Reno NV 89557, USA; 4 UCSF Library and Center for Knowledge Management, UCSF, 530 Parnassus Avenue, San Francisco, CA 94143-0840, USA

**Keywords:** Medical libraries, Medical informatics, Teaching, Active learning, Curriculum, Library science, Information science, Information literacy, Information fluency, Information seeking behavior

## Abstract

**Background:**

To conduct a systematic assessment of library and informatics training at accredited Western U.S. medical schools. To provide a structured description of core practices, detect trends through comparisons across institutions, and to identify innovative training approaches at the medical schools.

**Methods:**

Action research study pursued through three phases. The first phase used inductive analysis on reported library and informatics skills training via publicly-facing websites at accredited medical schools and the academic health sciences libraries serving those medical schools. Phase Two consisted of a survey of the librarians who provide this training to undergraduate medical education students at the Western U.S. medical schools. The survey revealed gaps in forming a complete picture of current practices, thereby generating additional questions that were answered through the Phase Three in-depth interviews.

**Results:**

Publicly-facing websites reviewed in Phase One offered uneven information about library and informatics training at Western U.S. medical schools. The Phase Two survey resulted in a 77% response rate. The survey produced a clearer picture of current practices of library and informatics training. The survey also determined the readiness of medical students to pass certain aspects of the United States Medical Licensure Exam. Most librarians interacted with medical school curricular leaders through either curricula committees or through individual contacts. Librarians averaged three (3) interventions for training within the four-year curricula with greatest emphasis upon the first and third years. Library/informatics training was integrated fully into the respective curricula in almost all cases. Most training involved active learning approaches, specifically within Problem-Based Learning or Evidence-Based Medicine contexts. The Phase Three interviews revealed that librarians are engaged with the medical schools' curricular leaders, they are respected for their knowledge and teaching skills, and that they need to continually adapt to changes in curricula.

**Conclusions:**

This study offers a long overdue, systematic view of current practices of library/informatics training at Western U.S. medical schools. Medical educators, particularly curricular leaders, will find opportunities in this study's results for more productive collaborations with the librarians responsible for library and informatics training at their medical schools.

## Background

Medical students must master skills to retrieve, critically assess, and integrate biomedical information into their clinical decision-making. These skills are recognized as core competencies. As Golub has noted, “The relatively short half-life of medical knowledge has led to the recognition of the importance of instilling the value and the skills of life-long learning as a core piece of medical education” [1]. Accordingly, over the past 75 years academic health sciences librarians have delivered information skills training as part of the formal education of medical students. William Dosité Postell, reporting on a survey conducted during the 1930s, indicated that 50 of the 64 medical schools in the U.S. (78%) offered library instruction [2]. Earl’s 1996 report on a survey of 123 academic health sciences libraries produced 55 responses with 75% reporting that they provided library instruction to medical students [3].

The 1982 Matheson Report advised educators that medical education in the future would bear little resemblance to the past due to a daunting expansion of medical information. Future physicians, while still in medical school, would need to acquire a new set of skills to manage and interpret the huge volume of information [4]. The Association of American Medical Colleges’ (AAMC) inventory of informatics competencies prompted some academic health sciences libraries in the U.S. to reassess, revamp, and redeploy their library instruction programs to better prepare medical students for a future requiring sophisticated information seeking skills. The arrival of these AAMC competencies generated a great deal of discussion among health sciences librarians, but it remained unclear as to the extent that librarians were ensuring that these AAMC competencies were integrated into medical school curricula [5,6].

Health sciences librarians perform a variety of expected and unexpected roles in U.S. medical school curricula, as validated by an extensive review of studies [7]. Health sciences librarians in the western U.S. have reported on a number of studies that focus on novel or effective library instruction approaches to training medical students at individual academic health sciences libraries [8-32]. No recent surveys have updated Earl’s 1996 study, however; and, there is an absence of research that reports comprehensively on the state of library instruction in the western region of the U.S.

Concerns about these research gaps drew the interest of a regional chapter of Libraries in Medical Education (LiME), an interest group of the Association of American Medical Colleges (AAMC) Group on Educational Affairs. LiME/AAMC meets annually as a means for members to report on current instruction related activities of librarians at institutions in the region. Wishing to take a more systematic and comprehensive approach, in 2009 a LiME research task group undertook an environmental scan of library instruction for medical students at all academic health sciences libraries serving accredited medical schools in the Western United States. The long term goal of the task force was to create a group of interested participants who could support a process of data gathering and reflection on current practices in order to improve the integration of library instruction into medical education. The purpose of this study was to facilitate broad comparisons between peer libraries by exploring in a comprehensive and systematic manner the ways in which academic health sciences libraries in the Western United States deliver instruction to medical students.

## Methods

The investigators implemented a three phase action research project consisting of (1) a descriptive environmental scan, (2) survey, and (3) interview methodologies. The present study included the common action research elements of researcher participation, real-life field settings, and reflective periods [33]. Vezzosi’s use of an action research approach to understand the effectiveness of library instruction represents a model of how action research can be employed in this subject area [34]. Somekh delineates eight principles normally found in action research in education contexts. The present study incorporated seven of those principles: a cyclical process; collaborative partnerships; knowledge development; roles of the researchers in the process; exploratory engagement; researchers as learners; and a broad contextual awareness [35].

### Phase one

Guided by discussion at LiME meetings and conversations between task force members, Phase One consisted of an unobtrusive environmental scan of publicly facing websites of academic health sciences libraries and educational institutions they serve, focusing on the 17 accredited medical schools of the Association of American Medical Colleges (AAMC) in the Western U.S. listed in Table 1. The investigators sought to construct a detailed picture of educational activities conducted by medical librarians and to identify common patterns of curricular support. Team members made preliminary investigations of public-facing websites at the institutions in the western U.S. Through an iterative process of review, reflection, synthesis, and discussion team members devised a checklist to apply to all 17 sites. This team-generated checklist guided reviewers in examining publicly-available documents such as library newsletters, course guides, and annual reports as well as relevant data from the Association of Academic Health Sciences Libraries (AAHSL) [36]. During the process, the investigators looked for unique or innovative library instruction practices. They also identified basic descriptive information about the user population of the library and, in some cases, information about the faculty status and committee appointments of library staff.

**Table 1:** Potential and actual participants in phases 1 & 2: academic libraries supporting schools of medicine

**University & Library**	**Responded to phase 2**
1. Charles Drew University of Medicine & Science, Health Sciences Library	✓
2. Loma Linda University Medical Center, Jesse Medical Library & Information Center	
3. Oregon Health and Science University, Library	✓
4. Stanford University Medical Center, Lane Medical Library	
5. University of Arizona (Tucson Campus), Arizona Health Sciences Library	✓
(University of Arizona (Phoenix Campus) Partnership of U of A & ASU medical school dissolved mid-project. ASU counted as part of U of A)	
6. University of California, Davis, Carlson Health Sciences Library	
7. University of California, Irvine, Science Library	✓
8. University of California, Los Angeles, Biomedical Library	✓
(University of California, Riverside program is developing, with most services provided by UCLA and therefore, counted under UCLA)	
9. University of California San Diego, Biomedical Library	✓
10. University of California, San Francisco, Library	✓
11. University of Colorado, Health Sciences Library	✓
12. University of Hawaii at Manoa, Health Sciences Library	
13. University of Nevada Reno, Savitt Medical Library	✓
14. University of New Mexico School of Medicine, Health Sciences Library and Informatics	✓
15. University of Southern California, Norris Medical Library	✓
16. University of Utah, Eccles Health Sciences Library	✓
17. University of Washington, Health Sciences Library	✓

Despite the variable quality and quantity of the initial results, Phase One provided useful information to help investigators articulate the following three research questions to guide phases two and three:

1. What are the current core or commonly followed practices of teaching library/informatics skills to medical students?

2. What patterns or possible trends might emerge from comparisons of different academic health sciences libraries in the Western US that provide library/informatics skills trainings for medical students?

3. What innovative practices can be identified at specific academic health sciences libraries that might be adapted to other academic health sciences libraries?

### Phase two

The team shared its analysis of the Phase one data with the larger LiME membership for comment and discussion to guide the design and distribution of a descriptive survey [37]. The survey’s final format incorporated the Phase One unobtrusive study data, the investigators’ own library instruction experiences, feedback from the (AAMC/LiME) group, and anecdotal knowledge of instructional activities typical in health sciences libraries.

The investigators designed the survey to learn: the medical school governance structure, the role (if any) of librarians in that governance structure, details about library instruction integrated within the curriculum, library instruction (if any) not integrated within the curriculum, faculty status, how library/informatics instruction skills were assessed, and a prediction as to whether graduating medical students at their institution would perform well on PubMed database searches on a United States Medical Licensure Exam (USMLE) currently under consideration by the National Board of Medical Examiners [38,39]. Additional file 1 contains the Phase Two survey questions.

The investigators secured Institutional Review Board approval (HRPO # 10–102) from the University of New Mexico to conduct the survey and any follow-up interviews in the last phase. The investigators deployed the invitation to complete the survey on April 7, 2010. The investigators emailed this invitation to the directors of all 17 academic health sciences libraries serving accredited medical schools in the Western U.S. as listed in the AAHSL Directory [40]. The directors were asked to forward the emailed invitation to all library employees responsible for conducting library instruction with medical students, as a modified form of snowball sampling. A total of three reminder emails were sent and the final invitation was sent in mid-June with an announced closing date of June 22, 2010. The invitation required all respondents to consent to participate in accordance with ethical research principles and invitees were asked to click on a link to the survey as their means of giving consent. Table 1 lists the institutions contacted with checkmarks aside those institutions responding to the survey. The investigators compiled the survey responses, discussed them at length via online conferencing software, and synthesized the data. In keeping with the reflective phase of action research, the results were shared with the librarian community in a panel presentation at a regional meeting of WGEA [41]. The ensuing commentary and discussion among meeting attendees were critical in devising the third phase of the study.

### Phase three

This phase of the project consisted of the investigators developing and deploying a standardized template of six (6) interview questions. The template additionally included some prompts intended to follow these specific questions so the interviewer might pursue any productive avenues for further discussion. The investigators interviewed the respondents at each institution who had the greatest breadth and depth of library instruction experience with medical students. The structured interview questions, and the prompts for possible follow-up, appear in Additional file 2. The investigators implemented the follow-up interviews lasting approximately 30 minutes each by telephone or online conference software beginning in December 2010 and completed the structured interviews during April 2011. All interviewees were sent summaries of the interviews so they might correct any responses, or add clarifying text.

## Results

This three-phase action research study produced results on the state of library/informatics training that can both inform current practices for medical educators and point toward future research. The environmental scan in Phase One generated targeted research questions about current practices while Phases Two and Three predominantly painted a picture of current practices.

### Phase one results

The information gathered from the websites ranged from ones that merely outlined the essential library services offered extending all the way to websites offering comprehensive accreditation self-study reports in accordance with the standards set by the Liaison Committee on Medical Education guidelines [42]. Inspection of the institutional websites revealed announcements of upcoming workshops, links to handouts from educational sessions and workshops, indications of curriculum-based courses, links to online multimedia tutorials, and access to supplementary instructional guides developed by librarians. While some of the institutions’ websites provided a complete picture of their library instruction activities, many lacked sufficient detail to accurately portray the roles that librarians play in supporting medical school curricula. The investigators recognize that some of this information might have been behind password protected websites and thereby unavailable. As noted earlier, the constraints of this purely descriptive approach resulted in an incomplete and inconclusive picture of library instructional programs. An analysis of gaps in the data helped to shape subsequent phases of the study and enabled investigators to generate targeted survey questions intended to yield comparative information about library instruction to medical students.

### Phase two results

Colleagues at 13 of 17 eligible academic health sciences libraries completed the survey, a response rate of 77%. Two librarians from one library completed the survey, and as their responses were consistent with one another, the investigators merged these responses. An informal follow up by one investigator with colleagues at three of the four non-responding libraries revealed that they did not have time to complete the survey. No significant geographic, governance, or other recognizable characteristics distinguished the non-responders from those who responded to the survey. For the responding libraries, all 13 medical schools governed their curricula with a curriculum committee. Academic health sciences librarians interacted with these curriculum committees directly through a variety of methods including regular membership, ex-officio membership, specialized subordinate groups, regular meetings with curricular leaders, or via informal contacts. The plurality of responses indicated that most organizations had multiple means of interaction but the primary method was via ex-officio membership on curriculum committees. A little more than half (53%) of the respondents had faculty status at their respective library and one also had an academic appointment through the school of medicine. All others had academic promotion systems equivalent to faculty status within their institutions. The respondents had an average of 18.4 years of experience as librarians and only three respondents had fewer than 10 years of experience. The majority of respondents had been involved in the most recent Liaison Committee on Medical Education accreditation review process.

**Figure 1:**
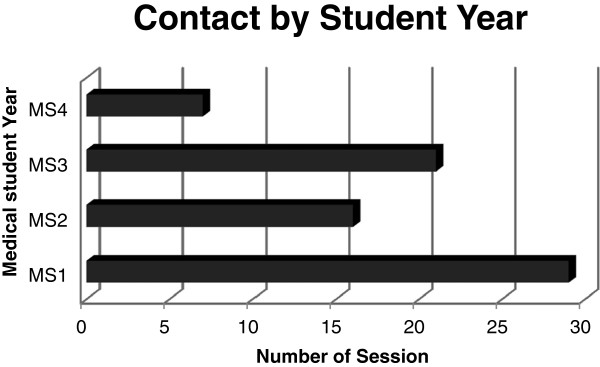
Library instruction by medical student year.

The Phase Two survey emphasized identifying instances where librarians engaged in curricular-based library interactions with medical students. All but one of the 13 institutions required incoming medical students to attend basic library orientation sessions. In total, 53 discrete sessions were described along with the year in which the students experienced the sessions. Responses showed activity occurred across the undergraduate curriculum. In general, librarians had an average of three (3) interventions integrated within the core curricula. Not surprising, first year medical students were the target audience for the majority of sessions (29 in total). Third year medical students were the second most frequently contacted audience (21 sessions) followed closely by the second year students (with 16 sessions). See Figure 1. Some sessions were composed of a mix of students from different years. The majority of sessions (*n* = 44) were required with less than 20% (*n* = 9 sessions) as elective sessions]. Fourth year student activity consisted primarily of liaison contacts or consults.

**Figure 2:**
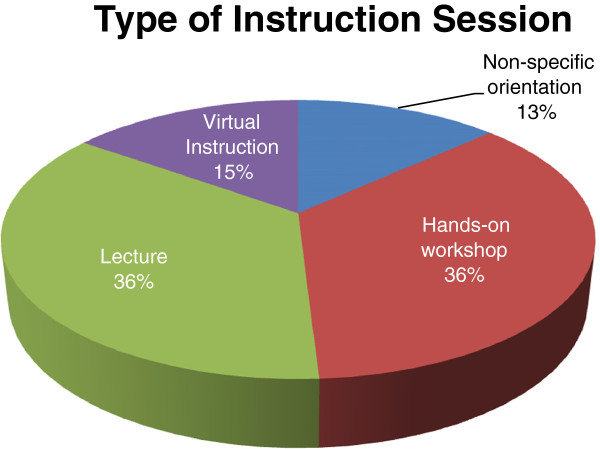
Types of librarian instruction.

Descriptions of the instruction sessions by respondents were consistent across the different institutions so that, even with institutional variances, responses could be categorized and quantified. Figure 2 summarizes the five (5) types of instruction sessions that emerged: hands-on, lecture, virtual, non-specific orientation, and required consults. Hands-on sessions included anything described with that term or a description indicating student interactions or student practice. Lecture sessions include those described as such as well as ones described as multiple week sessions. Hands-on sessions and lecture sessions were indicated equally with 19 sessions each. Virtual instruction is a growing trend in libraries [43] and the librarian medical educators noted 8 virtual instruction sessions which included work through blogs, online student peer assessment, wikis, videos, or online tutorials. Orientation sessions, not otherwise described, were left as such and termed non-specific orientation. See Table 2.

**Table 2:** Number of instruction sessions by format

**Format**	**Total**
Hands-on workshop	19
Lecture	19
Virtual Instruction	8
Non-specific orientation	7
Required Consult	1

Phase two results.

**Figure 3:**
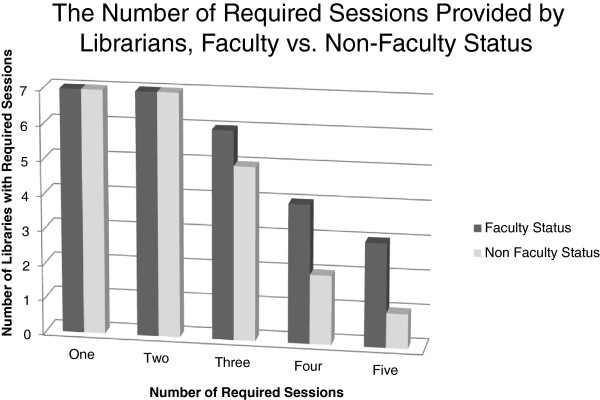
**Number of instruction sessions by librarian faculty vs. librarian academics, non-faculty status.** Faculty Status, Non-faculty status.

Other than ubiquitous PubMed sessions, two distinct topics were volunteered in the descriptions – evidence-based medicine (EBM) (23 sessions) and problem-based learning instruction (5 sessions). Figure 3 indicates that faculty status does not appear to have an impact on curriculum-integrated session *except* that faculty librarians tend to offer a few more required sessions (i.e., fourth and fifth sessions). Only a couple of the non-faculty librarians offered more than three sessions, and these were not always required. One-quarter of the descriptions voluntarily detailed time spent on instruction activities and future iterations of this study might request this specific information. For this small subset, the average time spent on instruction was 2 hours – ranging from a minimum of 30 minutes to a maximum of 32 hours (for a multi-week sequence).

Nearly one-quarter of the libraries reported an assessment of medical students prior to instruction. Formal assessment within the curriculum seems to be a rarely performed activity for librarians, however. As part of the curriculum, schools of medicine have some assessment of knowledge and skills, but it is unclear how the librarians are involved with that activity. Those responding to this question, representing 5 of the 13 schools, submitted just 10 instances of assessment. Yet, as a comparison, over 50 instruction sessions were entered in the survey. Of the assessments, a total of 9 were graded or pass/fail assignments with 2 having a self-assessment or peer-assessment component. Most of the described assessments involved activities such as finding resources and evaluating search skills in order to answer questions. Four sessions dealt specifically with evidence-based medicine (EBM) topics and only two specifically mentioned dealing with citations. The themes identified in Phase Two survey results are consistent with the literature in suggesting that medical students have a diminished preference for non-specific library orientations that lack a curricular context and focused learning objectives.

### Phase three results

During the autumn of 2010, the investigators held several in-depth online conference meetings to discuss the survey results. The structured interview questions to be used in Phase Three emerged from this action research process of review, reflection, and discussion. Twelve (12) of the 13 survey respondents were able to arrange interviews with an investigator during the allotted timeframe, a participation rate of 92%. The 12 interviews occurred during the December 2010 to April 2011 time period. The investigators engaged in both synchronous and asynchronous discussions to reach consensus on their interpretations of these interviews as summarized underneath each of the following italicized questions.

1. Could you explain the reasons for the successes you have experienced in integrating information literacy/fluency/competencies into your medical school's curriculum?

Answers varied widely, but some recurring themes emerged from the combined interviews:

• Librarians are engaged with the medical school curriculum committee and with curricular leaders.

• Librarians’ efforts frequently rely upon “champions” within the medical school who can advocate for integrating library/informatics skills.

• Librarians have strong support for library or informatics instruction from the library administration.

• Librarians have proven themselves to their teaching faculty colleagues or medical school administrators over time by demonstrating both their knowledge and teaching skills.

2. If we created a supplement to our upcoming article in a publicly accessible institutional repository that contains samples of outstanding handouts or other documents, would you be willing to contribute 3–5 of your best items?

• Responses point to a willingness to donate instruction related materials as well as enthusiasm for creating an open access archive.

3. Could you describe your online curricular or instructional support (examples: learning management system such as Blackboard; social networking; chat) at your institution? Does the library or another unit such as IT provide this support?

Most medical schools use a commercial learning management system. Most also use a locally produced learning management system to supplement the commercial system in order to meet all of their needs.

4. What were the "lessons learned" from past mistakes or miscalculations in your efforts?

• Free-standing courses never work as well as library instruction that is integrated fully into the curriculum

• The need to keep adapting to changing circumstances, including curricular changes, in the medical school

• Secure detailed feedback from students on the quality of teaching, its relevance to curricular content, and the content taught

• Perseverance despite setbacks usually leads to success

5. Why are librarians at your library motivated to teach?

Most librarians taught because of their faculty status, or were expected to teach due to a similar codified equivalent of faculty status as an institutional career ladder for promotion. Beyond this broad expectation, however, respondents noted that most librarians teach as a natural outgrowth of their desire to ensure that medical students (and future physicians) possess all needed library/informatics skills. One respondent mentioned that there were too few librarians to teach these skills on an individual point-of-use basis so formal instruction was the only reasonable cost-effective option. Interestingly, multiple authors made this central cost-effectiveness argument in a classic volume published in 1974 during a renaissance within library instruction in academic libraries [44]. Additionally, most respondents indicated that those librarians who teach certainly enjoy this instructional role.

6. Reviewing your responses concerning your activities, how much time was devoted to each?

Respondents’ formal work allocation to the education of medical students encompassed anywhere on average from 15 to 50% of their overall efforts. Most respondents reported that they spend a considerable amount of time outside of the classroom with curricular design, keeping abreast of curricular changes, and preparing to teach. On this latter point, one respondent mentioned spending 25 hours to perfect a presentation for a single one hour session in front of medical students since she realized that her time was so limited within today’s “crowded curriculum” [45] at US medical schools.

**Figure 4:**
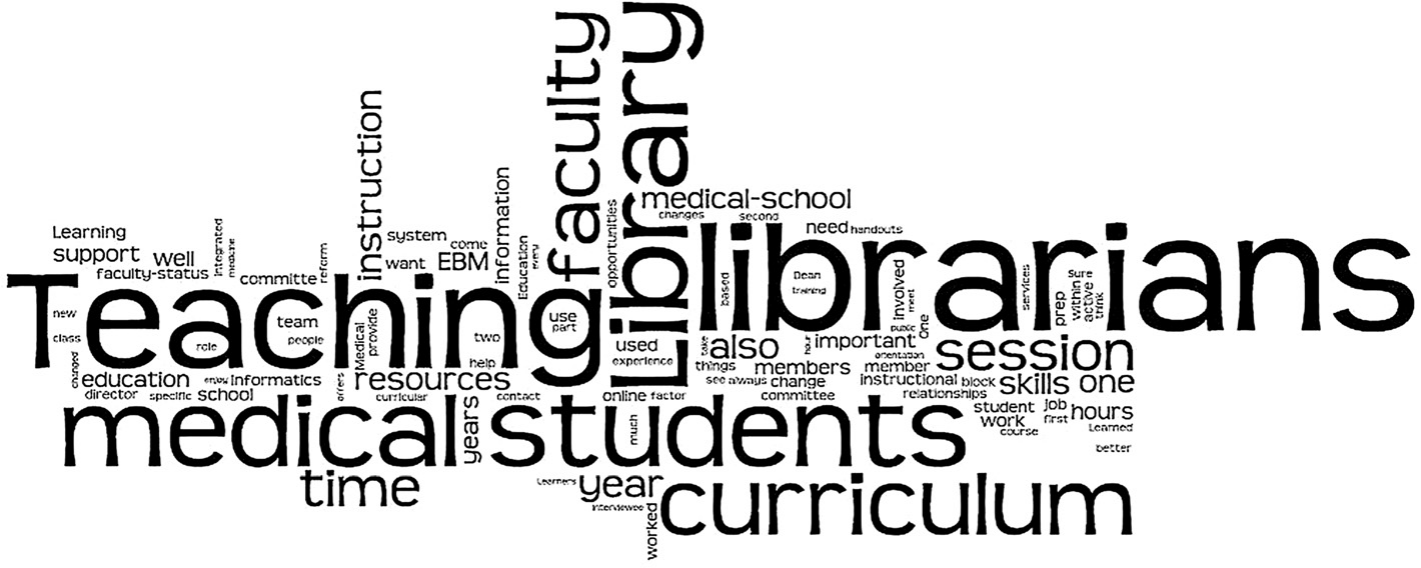
Wordle cloud from phase 3 interviews.

Figure 4 provides a Wordle™ word cloud that visually displays the words used most frequently by interview respondents. The investigators expected to find words such as “librarians” and “library” prominently displayed in the word cloud. The investigators did not expect to see the words “teaching”, “medical students”, “curriculum”, or “faculty” so frequently mentioned. Thus, the word cloud discovered some less obvious patterns otherwise lost by reading the texts of the structured interviews compiled in Phase Three.

## Discussion

This study fills a gap in health sciences library/informatics skills training at different US medical schools. The investigators discovered that many of their colleagues achieved success in integrating library and informatics skills into their respective curricula. This project readily confirmed the diversity of practices. This study also produced suggestive non-statistical evidence for librarians’ status and roles in curricular governance.

### An action research approach

Consistent with the tenets of an action research approach, the investigators followed an evolutionary, developmental course in order to better explore the challenges facing library/informatics instructors in medical education. By examining and sharing data in a stepwise approach, investigators were able to integrate discussion and concerns from the practitioner community in order to improve each subsequent phase of inquiry. This iterative process of engagement contributed significantly to the intended goal of producing a useful report on practices and trends in library instruction.

Because the investigators were members of the very AAMC/LiME community of practitioners under study, they bridged conventional forms of dichotomy between themselves and their subjects, contributing to a collaborative co-construction of knowledge [33]. Incorporation of key aspects of action research in the study, including building relationships, acknowledging and sharing power, and encouraging participation of the study population [34], enhanced the eventual applicability of results to professional practice.

### Phase one

This phase revealed that an institution’s publicly facing website cannot be relied upon to gather enough data to make more than just superficial comparisons across institutions on library education. The investigators learned in this process moreover that the availability of more robust data would be inconsistent across institutions, at best.

### Phase two

The survey addressed many of the investigators’ questions generated during Phase One. A particular focus examined the extent to which curriculum integration is reflected in library instructional activities. The literature has long suggested that increased educational effectiveness and impact on student learning is predicated on integration of library instruction into the existing medical curriculum, rather than as a separate component of a library’s educational program [46].

In her landmark article, Francesca Allegri defines “course integrated instruction” as having met at least three of the four following criteria: “(1) faculty outside the library are involved in the design, execution, and evaluation of the program, (2) the instruction is curriculum-based, in other words, directly related to the students’ course work and/or assignments, (3) students are required to participate, and (4) the students’ work is graded or credit is received for participation [47].” Survey responses reflected and met Allegri’s definition of course integrated instruction. Respondents described a variety of curriculum integration activities such as recurring roles in semester long classes; collaborative teaching of informatics concepts to support problem-based learning exercises, and interactive instruction covering content tied directly to exam questions. EBM training has evolved over the past few decades with librarians having a growing role in working with both students and faculty within the curriculum [48,49]. The survey responses indicated that over 40% of the sessions were EBM topics, a finding that validates much of the research in this area.

Faculty status or its close equivalent for librarians appears to provide access and credibility for librarians needing to integrate library or informatics training into medical school curricula. Librarians and teaching faculty members alike seemed to recognize their mutual interdependence in these endeavors. One of the founders of the modern library instruction movement, Evan Farber, has emphasized this mutually-dependent relationship between librarians and their teaching faculty colleagues [50]. Travis has admonished her colleagues more recently that “Librarians need to think and act globally, never compartmentalize library instruction efforts, and find ways to scale information literacy into an institution wide model [51].” Librarians at the institutions in this study apparently were paralleling Travis’ advice as further evidenced by their successes. Librarians involved in providing integrated library and informatics instruction had an average of 18 years’ experience, which strongly suggests that this role requires considerable experience, knowledge, and expertise. Wiggins similarly has noted that library or informatics instruction often succeeds when the experienced and knowledgeable librarian can provide skeptical students with the rationales for the relevance of library instruction at a specific juncture in the curriculum [52]. The recent resurgence of interest among educators on the national level in cultivating affective educational objectives also dovetails with this data [53].

### Phase three

The Phase Three interviews highlighted the importance of having champions among the teaching faculty and the support of administrators overseeing the curriculum. Curzon has emphasized the importance of such partnerships, particularly with teaching faculty who must balance a crowded curriculum with the student’s escalating need to effectively manage the exploding information universe [54]. In the absence of a context or perceived need among the students, interview respondents reported that the basic library orientation sessions tend to have poor educational outcomes. Prior research had suggested that library/informatics instruction most likely will be more effective when integrated into the curriculum [55]. This study preceded publication of Moore’s 2011 sentinel *Academic Medicine* commentary on the need for library/informatics training. The findings in this study provide supplementary evidence to support Moore’s thesis [56] as well as revealed innovative ways librarians are maximizing limited instruction time with their curricular partners.

### Limitations

This study details an environmental scan that explored the breadth and depth of library/informatics skills instruction for medical students at academic health sciences libraries in the Western U.S., and represents a unique examination of a largely uncharted subject area. The authors could identify only one account that bore even a distant similarity to the approach found in the present study [57]. The research reported in this present study cannot be generalized to the entire U.S. due to the geographic concentration in the western region, the small number of institutions, and the investigators’ awareness of diverse library instruction practices in other regions. The survey responses also constituted low-level frequency and descriptive data that could not be easily categorized into discrete data points. Still, medical educators and librarians outside the region can benefit from learning about the rich and diverse descriptive information on how their colleagues at different western U.S. institutions grapple with challenges similar to their own. In the process of implementing this three phase action research project the investigators have created a template for a national level action research study. This template could even be modified to secure more defined responses, if viewed by colleagues elsewhere as desirable. The investigators would be happy to share with interested colleagues their experiences in conducting this type of multiple methods study.

### Future research

Expanding the focus of this research beyond the Western region would provide a sufficient sample of librarians to make statistically significant test of the following hypotheses:

1. Great diversity in how medical students are trained on library/informatics skills exists in the United States, and that knowledge of some of these practices will be valued by colleagues involved in similar types of library/informatics training.

2. A correlation exists between librarian roles in governance structures *and* their degree of involvement in training medical students on library/informatics skills, the degree to which this training has been integrated into the curriculum, and their assessment of medical student performance.

## Conclusion

This study provides medical educators and librarians with a detailed snapshot illustrating the current nature of library instruction in medical schools. It delineates the degree to which these library/informatics competencies are integrated into medical school curricula. Analysis of the information examines some preconditions for successful instructional programs, reveals challenges shared by librarian instructors, and discusses adaptive strategies that have led to greater student satisfaction. The results reinforce the notion that information skills instruction is an important part of medical education and are indicative of the value librarians contribute to the educational process.

Medical educators, if not already doing so, should actively partner with librarians at their institution to strive for curriculum integrated information skills training of medical students. Librarians should also ensure that feedback on library instruction is included as a standard component of student course evaluations. Folding evaluations of library instruction into the broader curricular context may increase the validity of student feedback, give instructors meaningful data with which to quantify skills improvement, enhance future library instruction, and relieve students of the burden of completing separate post-instruction library surveys. Librarians play a pivotal role in providing the skills to bolster life-long learning that goes well beyond medical school and prepares a solid foundation for how to keep up with the ever-growing body of medical education research literature.

## Competing interests

The authors declare that they have no competing interests.

## Authors’ contributions

JE, KH, TH, and JT conceived of this project. JE developed the design and secured IRB approval and subsequent renewals. JE, KH, TH, and JT conducted the Phase One environmental scan and designed the Phase Two survey. KH implemented the survey via SurveyMonkey™ and codified and tabulated the results. JE, KH, and TH interpreted the survey results and designed the follow-up interview questions in Phase Three. JE, KH, TH, and JT conducted the in-depth interviews in Phase Three. JE, KH, TH, and JT wrote and edited the manuscript. All authors read and approved the final manuscript.

## Pre-publication history

The pre-publication history for this paper can be accessed here:

http://www.biomedcentral.com/1472-6920/13/119/prepub

## Supplementary Material

Additional file 1 Phase Two Online Survey Questions. Click here for file

Additional file 2 In-Depth Phone Interview Questions. Click here for file
