# Supplementary material for: Current practices in library/informatics instruction in academic libraries serving medical schools in the western United States: a three-phase action research study
Source: BMC Med Educ. 2013 Sep 4;13:119. doi: 10.1186/1472-6920-13-119 (PMC3847693; doi:10.1186/1472-6920-13-119)
Supplement: Additional file 1 — Phase Two Online Survey Questions. [file 1472-6920-13-119-S1.docx]

**Additional File 1**

**Phase Two: Online Survey Questions (done in SurveyMonkey)**

1. How is the medical school curriculum governed at your institution? (Check all that apply)

Curriculum committee or equivalent

Dean (s) of education

Departmental chairs

Other (please explain)

2. How do librarians at your institution communicate with those groups or individuals who govern the curriculum? (Check all that apply)

Voting membership(s) in decision making group(s)

Ex Officio (non-voting) membership in decision making group(s)

Standing meeting(s) with curricular leader(s)

Other (please explain)

3. List the year(s) of medical school (1st, 2nd, 3rd, 4th) and in what contexts library/informatics skills are covered in the curriculum. For each instance, indicate if these skills are elective, required, or linked to successfully completing a required activity. We have provided an example below.

Example of information needed.


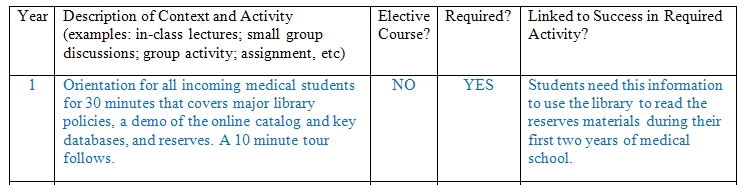


We have provided space for up to 5 classes below. If you need more, please contact us.

4. List the year(s) of medical school (1st, 2nd, 3rd, 4th or ongoing/recuring) and in what contexts library/informatics skills are covered OUTSIDE the curriculum.

Example of classes outside the curriculum.


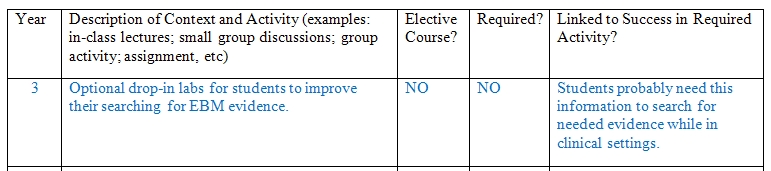


We have provided space for up to 5 classes below. If you need more, please contact us.

5. If librarians at your institution are not included in the medical school curriculum, but have created online resources (e.g., tutorials, libguides, etc.) intended to serve medical students, please describe these efforts and paste any publicly available links.

6. Does the library or the medical school assess the library/informatics skills of all incoming students?

Yes/No

7. Please list below all instances in the medical school curriculum when students are formally assessed on their library/informatics knowledge or skills. If librarians do not teach these skills formally, please indicate who provides this assessment.

If assessment information is unknown, please comment.

Assessment example


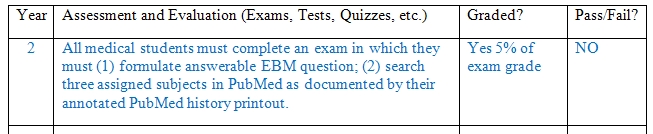


We have provided space for up to 4 instances of assessment. If you have more instances, please contact us.

8. If the USMLE exam in the future were to include testing students on their abilities to search subjects in a database such as PubMed, do you think that graduates of and the current students in your medical school would have received sufficient training to perform well on such a text segment?

Yes/No

Please explain:

9. Have the librarians at your institution been involved in the preparation of an LCME self-study or site visit within the past four years?

Yes/No

If so, would it be possible to obtain a copy of the associated report to this survey?

10. We would like to survey and communicate with all librarians at your institution that might be involved in teaching medical students.

I have no colleagues involved in educating medical students.

I have one (or more) colleagues involved. Their contact information is below.

11. Please list all librarians or library staff members who play roles in medical students’ knowledge of and skills in library/informatics subjects. We have space for you to add up to five colleagues.

12. Please let us know your ...

Year's of Health Science Librarian Experience

Gender

13. Do you have faculty status at your institution?

Yes/No

Other (please specify)

14. Would you like to have your institution credited for any innovative practices that we choose to highlight in our report?

Yes/No

15. We would like to contact you for a follow-up interview within the next few weeks. If you are willing to participate further, please provide your contact information.
