# Supplementary material for: Current practices in library/informatics instruction in academic libraries serving medical schools in the western United States: a three-phase action research study
Source: BMC Med Educ. 2013 Sep 4;13:119. doi: 10.1186/1472-6920-13-119 (PMC3847693; doi:10.1186/1472-6920-13-119)
Supplement: Additional file 2 — In-Depth Phone Interview Questions. [file 1472-6920-13-119-S2.docx]

**Additional File 2**

**Phase Three: In-Depth Phone Interviews: Standardized Questions**

**With Interviewer Optional Prompts**

1. Could you explain the reasons for the successes you have experienced in integrating information literacy/fluency competencies into your medical school's curriculum?

**Possible Interviewer Follow-up Prompts on contributory factors**

Curricular change?

Champions of library instruction within the institution?

Librarians with backgrounds in teaching, instructional design, or other education expertise?

Librarians with faculty status? Or, librarian appointments to curricular or other committees?

2. If we created a supplement to our upcoming article in a publicly accessible institutional repository that contains sample outstanding handouts or other documents, would you be willing to contribute 3-5 of your best items?

3. Could you describe your online curricular/instructional support (examples: learning management system such as Blackboard; social networking; chat) at your institution? Does the library or another unit such as IT provide this support?

**Possible Interviewer Follow-up Prompts:**

Review their responses so they are certain they have included everything

4. What were the "lessons learned" from past mistakes or miscalculations in your efforts?

5. Why are librarians at your library motivated to teach?

6. Reviewing your responses concerning your activities, how much time was devoted to each?

7. Explain that we will briefly summarize each interview and allow interviewee to review the summary.
